# Atherosclerosis Induced by *Chlamydophila pneumoniae*: A Controversial Theory

**DOI:** 10.1155/2013/941392

**Published:** 2013-07-17

**Authors:** Hamidreza Honarmand

**Affiliations:** ^1^Department of Microbiology, Guilan University of Medical Sciences, Iran; ^2^Heart Research Center, Guilan University of Medical Sciences, Iran

## Abstract

More than a century ago, inflammation and infection were considered to have atherogenic effects. The old idea that coronary heart disease (CHD) possibly has an infectious etiology has only reemerged in recent years. Atherosclerosis is the main pathological process involved in CHD and is, logically, the first place to look for infectious etiology. The process of atherosclerosis itself provides the first hints of potential infectious cause. Smooth muscle proliferation, with subsequent intimal thickening, luminal narrowing, and endothelial degeneration, constitutes the natural history of atherosclerosis, being with the severity and speed of these changes. Both viral and bacterial pathogens have been proposed to be associated with the inflammatory changes found in atherosclerosis. Recently, *Chlamydophila pneumoniae* (*C. pneumoniae*) has been implicated as a possible etiologic agent of coronary artery disease and atherosclerosis. New evidence which supports a role for *C. pneumoniae* in the pathogenesis of atherosclerosis has emerged. *C. pneumoniae* has been detected in atherosclerotic arteries by several techniques, and the organism has been isolated from both coronary and carotid atheromas. Recent animal models have suggested that *C. pneumoniae* is capable of inducing atherosclerosis in both rabbit and mouse models of atherosclerosis. Furthermore, human clinical treatment studies which examined the use of antichlamydial macrolide antibiotics in patients with coronary atherosclerosis have been carried out. The causal relationship has not yet been proven, but ongoing large intervention trials and research on pathogenetic mechanisms may lead to the use of antimicrobial agents in the treatment of CHD in the future.

## 1. Introduction

Cardiovascular disease, resulting from atherosclerosis, is a leading cause of global morbidity and mortality [1]. Despite the tremendous gains made in decreasing the number of deaths due to cardiovascular disease, it still is health care's greatest challenge [2]. Genetic predisposition and classical environmental risk factors (family history, hypercholesterolemia, cigarette smoking, hypertension, diabetes, obesity and excessive drinking all figures into the equation) explain much of the attributable risk for cardiovascular events in populations, but other risk factors for the development and progression of atherosclerosis, which can be identified and modified, may be important therapeutic targets [1].

Traditional risk factors account for about 50% of the incidence of cardiac disease. In fact, many individuals who develop heart disease have normal cholesterol and blood pressure levels. This suggests that other less well-studied risk factors may also play a role [2]. Furthermore, exact knowledge regarding the mechanisms by which the various established risk factors contribute to the development and progression of lesions is incomplete. These facts have led investigators to pursue other possible etiologies and factors that may be involved in the etiology and pathogenesis of atherosclerosis and its complications [3].

Actually atherosclerosis is a complex, multifactorial disease. Recently, research has intensified to identify the role of various infections in the pathogenesis of atherosclerosis and the complications, such as ischemic heart disease and stroke. Examination of an atherosclerotic plaque reveals pools of cholesterol under a fibrous cap and the infiltration of monocytes and T cells at its margins. This concentration of white blood cells within the plaque is consistent with an ongoing inflammatory process, influenced by factors not yet fully understood. One such influence may be infection. Specific agents have been proposed as direct initiators or accelerators of atherosclerosis, while other infectious agents have been proposed as accelerators of atherosclerosis through nonspecific stimulation of the inflammatory cascade. Recently, the total pathogen burden concept has suggested that while each specific infection contributes only slightly to the pathogenesis of atherosclerosis, the cumulative effects of infectious agents contribute greatly [4].

Now researchers are theorizing that infection may be responsible for at least some cases of heart disease. What they are talking about is not an overt, active attack as with a cold or the flu but rather a chronic, low-grade infection that may linger for years barely noticeable. The idea surfaced in 1988 when a study published in the British medical journal The Lancet observed that the bacterium *Chlamydia pneumonia* (now* Chlamydophila pneumonia*) was frequently present in artery-clogging deposits. Other studies found that men with gum disease and similar infections had a higher rate of heart attacks. No conclusive cause/effect relationship was proven then, nor has one been found since. Nevertheless, a large number of studies over the past decades have found a strong correlation that requires further study [5].

Microorganisms can find their route to heart system directly. The second route by which infection may result in progression or initiation of an atherosclerotic lesion involves the dissemination of organisms from local sites of infection directly to the arterial wall itself. The organisms may traffic to the site within an infected monocyte, attach and then diapedesis through the endothelial cell layer, taking advantage of secondary host defense mechanisms to infect distal tissue. Once at the site, the organisms could drive a local inflammatory process or, in addition, infect other cells within the arterial wall.

In fact the hypothesis that infectious agents are causal agents in atherosclerosis originally was formulated in the first two decades of last century [6]. However, this concept received little attention until the late 1970s, when Fabricant et al. showed that chickens experimentally infected with an avian herpes virus developed florid vascular lesions similar to those of human atherosclerosis. Subsequently, many investigators have reported observations implicating certain infectious agents in human atherosclerotic disease. Two basic lines of evidence have been presented: (1) detection of the agent in atherosclerotic lesions by immunocytochemistry and molecular biology and (2) epidemiological evidence based on serological data implicating an association between atherosclerotic disease and positive serology [6, 7].

Actually microbes may potentially play a role in the development of chronic artery disease (CAD) at several steps: (i) initial endothelial vascular injury via direct or indirect mechanisms that could initiate atherosclerosis; (ii) acceleration of early atherosclerosis via local increase in LDL and oxidized LDL by cytokines stimulation or by predisposing to abnormal lipid profile; (iii) precipitation of acute events by predisposing to vulnerable plaque or activation of the coagulation cascade [6].

## 2. Bacteriology 

*Chlamydophia pneumoniae*, an intracellular bacterium of the Chlamydiaceae family, is a common respiratory pathogen causing community-acquired pneumonia, bronchitis, sinusitis, and upper respiratory tract symptoms. It is known as a leading cause of human respiratory tract infections worldwide. The infection is frequently mild and clinically unapparent, and its prevalence in the population with increasing age almost mirrors the prevalence and extent of atherosclerosis [8]. This organism is spread easily through airborne droplets and can linger for years inside body cells without causing noticeable symptoms. *C. pneumonia* is an obligate, intracellular, gram-negative bacterium that is distinguished from other bacteria by a unique growth cycle. In this growth cycle there are 2 morphologically and functionally distinct cell types: the infectious elementary body (EB) and the reproductive reticulate body (RB). Chlamydia exists as elementary bodies in the environment. Upon entry into a host cell the elementary body undergoes a series of transformations that allow it ultimately to replicate. At this stage it is referred to as a reticulate body. After cell division, it again reverts to an elementary body and is released from the host cell. If, however, host cell conditions are not favorable, Chlamydia will not progress through cell division and instead moves into what has been referred to as a persistent state, appearing morphologically as a large, aberrant form.

The outer envelope of the elementary body is composed of cysteine-rich structural proteins with molecular masses of 98, 60-doublet, 39.5, and 15.5 kDa*. C. pneumoniae *infection is rare before 5 years of age, but by 20 years 50% of people have antibodies, and by 65 years more than 80% have been infected [9]. Outer membrane protein 2 (Omp2) is a second constituent of the chlamydial outer membrane complex; it has a molecular mass of ′60 kDa. Omp2 has been found to be a major immunogen of *C. pneumoniae. *Yet another 60-kDa protein has been identified in *C pneumoniae*; it is a homolog of *C. trachomatis *GroEL and a member of the heat shock protein family, HSP60. *C. pneumoniae *strains are able to multiply within macrophages, where they persist for long periods without causing any damage until they are reactivated by immunosuppression or by coincidental infection with other organisms.

In order to play a causative role in chronic disease, *C. pneumoniae *would need to persist within infected tissue for extended periods of time, thereby stimulating a chronic inflammatory response. *C. pneumoniae *has been shown to disseminate systemically from the lungs through infected peripheral blood mononuclear cells and to localize in arteries where it may infect endothelial cells, vascular smooth muscle cells, monocytes/macrophages and promote inflammatory atherogenous process [10]. Chlamydiae is notorious for causing chronic infections, and treatment failure is common. These infections and failures may be due to the establishment of a nonreplicating and noncultivable growth stage but viable state of Chlamydiae resulting in a long-term relationship with the infected host cell. Atherosclerotic disease progression is associated with higher levels of antibodies due to chronic infection, repeated infections, enhanced antigen presentation, or any combination.

### 2.1. Lab Experiments

Numerous studies have found evidence of *C. pneumoniae *in arterial plaques by several different techniques (immunehistochemistry, in situ hybridization, PCR, electron microscopy, and cultures) but rarely in normal arteries [6]. The mean prevalence of *C. pneumoniae *in atherosclerotic plaques is about 40–50%, and the presence of the organisms in CAD or other arteries do not correlate with the standard antibodies measured in clinical trials [6, 11]. Hence recent randomized control trials (RCTs) that determine inclusion of patients by antichlamydial antibodies or the assumption that most patients have chronic persistent *C. pneumoniae *infection are likely to be underpowered.

### 2.2. Animal Model

Animal experiments in rabbits have demonstrated that *C. pneumoniae *can initiate early atherosclerotic changes in the aorta without hyperlipidemia [12]. However, the majority of animal experiments have demonstrated that *C. pneumoniae *and *P. gingivalis *can accelerate atherosclerosis in the presence of hyperlipidemia [13]. Although theoretically these microbes could lead to precipitation of acute cardiac events by destabilization of plaques through induction of metalloproteinases or gelatinase by macrophages [14, 15], or by inducing tissue factor to cause or precipitate acute thrombosis (clot) [16], no animal models have shown this effect. Thus, the recent randomized clinical trials (RCTs) were designed to prevent secondary cardiac events (or precipitation by chronic *C. pneumoniae *infection) which have never been demonstrated to occur in animal models.

Muhlestein et al. [17] reported that in the rabbit model (fed cholesterol enriched diet) azithromycin could prevent acceleration or enhancement of atherosclerosis (when treated immediately after infection), and delayed treatment has been shown to be ineffective in preventing artherosclerotic changes [18]. Furthermore, there is evidence that single agents (such as azithromycin or ofloxacin) cannot eradicate *C. pneumoniae *in a chronic persistent state in either a continuous cell culture model or experimental murine pneumonitis model [19, 20]. Hence the antibiotics used in the recent RCTs are not very effective in eradicating *C. pneumoniae *from tissues in animals or even within human monocytes [21]. Thus the negative results from these RCTs could be secondary to an ineffective regimen. Currently the best choice of agents to eradicate persistent chronic infection with *C. pneumoniae *is unknown but may be a combination with rifampin [20].

Other factors that could affect the outcome of these negative RCTs include the fact that the majority of patients were already receiving optimal therapy, such as selective *β*-blocker, antiplatelet drugs (i.e., aspirin) and “statins.” thus making it extremely difficult for any additional therapy to show any difference above standard treatment. This also applies to lack of benefit seen with HDL-modifying agents. Moreover it has been demonstrated *in vitro* that “statins” can inhibit *C. pneumoniae *in cell culture and suppress the inflammatory or cytokine response to this microbe that may be playing a role in atherogenesis [22]. Recent studies demonstrate that simvastatin reduces *C. pneumoniae*-mediated histone modification and gene expression in cultured endothelial cells, thus downregulating cytokine production that are important in initiating and accelerating atherosclerosis [23]. Furthermore in acute infection of swine with *C. pneumoniae *via the respiratory tract, there is impairment of the muscarinic and kinin-related reactivity of coronary circulation, which can be prevented by simvastatin [24].

In addition, evidence that *C. pneumoniae *can either initiate or accelerate the atherosclerotic lesion has come from work with both mice (NIH/s, ApoE-deficient, and LDL-receptor knockout strains) and New Zealand white rabbits. These animals generally need to consume a high cholesterol diet in order to develop observable changes, though it is possible, in one of the rabbit models, to observe effects without an atherogenic diet [18]. In the LDL receptor knockout mouse, intranasal inoculation with the *C. pneumoniae *AR39 strain twice monthly for six months was performed prior to sacrifice of the animals. Uninfected mice fed a high cholesterol diet had a lesion area index (defined as the size of a digitized image of the lesion divided by the aorta luminal surface and multiplied by one hundred) of 18, while infected animals given a high cholesterol diet had an index of 42. This 130 percent increase in lesion size suggests that infection with *C. pneumoniae *can accelerate the growth of an atherosclerotic plaque [25].

There are limitations to the interpretation of animal models of atherosclerosis. In some of these models the atherosclerotic lesions observed are consistent with a very early pathologic process that does not mirror the lesions responsible for causing human disease. The atherosclerotic lesions in these models generally do not rupture or lead to clinical disease in the animal. While these data do support the potential for a contribution of *C. pneumoniae *to lipid accumulation at the site, they do not provide conclusive evidence that infection will lead to plaque rupture.

## 3. Seroepidemiological Studies

Seroepidemiological studies have shown an increasing prevalence of antichlamydial antibody with age, indicating the presence of acute *C. pneumoniae *infection beginning in childhood and extending to adulthood. The prevalence increases from ages 5 through 14 years, and by age 20 years, approximately 50% of persons have serum antibodies to *C. pneumoniae*. The seroprevalence continues to increase, reaching 80% in the elderly. The data suggest that most people are infected and reinfected throughout life.

Since the initial study that identified an association between elevated *C. pneumoniae *antibody titers and the prevalence of coronary artery disease, over thirty additional studies have been performed. These studies used different antibody detection assays with different titer cutoffs, different case definitions of coronary artery disease, and were performed in different geographic regions. Overall, it appears that elevated antibody titers to *C. pneumoniae *are associated with a threefold increase in the likelihood of having coronary artery disease. The association identified in seroepidemiological studies using titers to predict the incidence, distinct from the prevalence, of heart disease, however, only variably detects an association and, when positive, only in the range of a 20–40 percent increased risk [26]. While the implications of these different findings are being evaluated, the main value of these seroepidemiologic studies may be the attention they have brought to the potential for any association at all.

Most epidemiological studies of *C. pneumonia *antibodies and CAD or cerebrovascular disease reviewed by Danesh and colleagues that reported a 2-fold or larger odds ratio. Potential shortcomings of these data are the different end points used to define seropositivity and the subjectivity of interpretation of the microimmunofluorescence assay. Although some of the studies were small and may have had statistical biases for subgroups, in general the total of 2700 cases supported the existence of an association between *C. pneumoniae *and CAD [3].

Since the review by Danesh and colleagues, [27] there have been several relatively small case-control series that have shown the same association [28–32]. However, 3 recent studies warrant further mention. In a relatively large case-control study, the presence of antibodies to *C. pneumonia *was found to be associated with stroke or transient cerebral ischemia [33]. A large prospective longitudinal study over 13 years showed independent correlation of IgA antibodies, but not IgG antibodies, to *C. pneumonia *with excess mortality from CAD [34]. A smaller prospective study over 12 years showed no correlation between *C. pneumoniae *IgG seropositivity and future myocardial infarction or C-reactive protein; there were no tests for IgA [35]. Chronic *C. pneumoniae *infection itself may be associated with a serum lipid profile that predisposes to atherosclerosis [36].

## 4. Histopathology 

The next series of studies involve histopathologic examinations of the atheromatous plaque. In the first 15 studies reported in the literature which were conducted in the United States and Europe, approximately 45 percent of the total of 574 samples examined was found to contain evidence of *C. pneumoniae *by either immunohistochemistry, electron microscopy, *in situ *polymerase chain reaction (PCR) or, rarely, culture. The primary criticism of these studies has focused on the lack of standardization of the assay techniques but, given the bulk of the observations from these and subsequent studies, it seems likely that this pathogen can be found in the plaque.

## 5. Polymerase Chain Reaction (PCR)

Because antibody titers merely suggest historical exposure to the pathogen, there has been a recent interest in the use of PCR to identify individuals that may have an active infection with *C. pneumoniae*. PCR has been used to assess both histopathologic specimens and circulating white blood cells. In four published papers, patients with a history of coronary artery disease were more likely than controls to have *C. pneumoniae *identified in circulating monocytes by PCR [26]. In a fifth paper, the incidence was not significantly different but the *C. pneumoniae *rRNA copy number was higher in patients with heart disease [37]. Of interest, the proportion of individuals with PCR positive cells in these studies ranged from 9 to 60 percent in the patients with heart disease and 2 to 46 percent in the controls. While this range of exposure may be explained by epidemiologic influences, technical concerns about assay methodologies remain and efforts at standardization have been initiated [38]. When the technical concerns have been addressed, it will also be important to understand why otherwise normal individuals have evidence of this pathogen circulating in what should be a sterile space.

## 6. Culture 

*C. pneumoniae *is difficult to culture from atheromatous tissue and has been recovered on only a few occasions. The current theory for the difficulty in culturing the organism from atheromas is that it is residing in a latent, persistent state with low metabolic activity associated with “uncultivable forms” of *Chlamydia*.

### 6.1. Clinical Trial with Antibiotic

Preliminary trials of antibiotic therapy for secondary prevention of cardiovascular events suggested a benefit of newer macrolides after acute myocardial infarction or in unstable angina [39, 40]. However, in the larger randomized study, the reduction in secondary events observed at 1 month after treatment was lost at 6 months' followup [41]. In another relatively small randomized study involving patients with CAD (previous myocardial infarction, coronary artery bypass surgery or more than 50% stenosis of one or more major coronary arteries) who were seropositive for *C. pneumoniae*, no reduction in secondary cardiovascular events was present 6 months after a 3-month regimen with azithromycin [41]; however, there was reduction of a global rank sum score of 4 inflammatory markers (C-reactive protein, interleukin-1, interleukin-6, and TNF-*α*) in the treated group.

### 6.2. New Experiments

To further establish this relationship would need more specific and sensitive diagnostic blood tests that reflect chronic persistent infection and correlate well with the presence of *C. pneumoniae *in atheromatous plaques. One potential candidate for diagnosis of persistent *C. pneumoniae *infection is a recently developed enzyme immunoassay [EIA] for quantification of chlamydial LPS in serum [42]. However, combined serological and pathological studies need to be performed to confirm the correlation of the cLPS and presence of the organisms in atheromatous plaques. Another potential candidate diagnostic test is determination of chlamydial heat shock protein (cHSP) antibody [43]. However, only one small study to date has been performed to determine correlation with pathological evidence of *C. pneumoniae *in atheromas [11]. Commercialization of Hsp-60 monoclonal antibody (Genway Biotech) has now paved the way for further larger histopathogical-serological studies by other centers. This is crucial before undertaking any further large prospective epidemiological cohort studies or RCTs of any therapeutic agent.

A key issue in the hypothesis of *C. pneumoniae *role in atherogenesis is the ability of this microbe to persist intracellularly as an aberrant body, which has been mainly described in chronic *Chlamydia trachomatis *infections [44]. These persistent aberrant forms are in an arrested state in development, nonculturable, and explain the low yield of culturing human atheromas for *C. pneumoniae *[6]. In a recent study the presence of aberrant or persistent bodies of *C. pneumoniae *were demonstrated in human coronary atheromas by immunogold electron microscopy technique [45]. To define the serological responses to *C. pneumoniae *antigens that are associated with persistent infection, another recent report described antibody patterns of sera from subjects with and without evidence for persistent *C. pneumoniae *(determined by multiple PCR analysis at different times of peripheral blood mononuclear cells and vasculatory samples) by using proteomics, combined with 2D gel immunoblotting [46]. In this study a unique antibody response pattern (by differential reactivity for 12 proteins) were found which reflected persistent *C. pneumoniae *and was not predicted by the current gold standard for serodiagnosis (the immunofluorescence test). The method used in this report, however, would be too expensive, time consuming, and cumbersome to be used for large multicenter population studies and clinical trials. Thus application of this data to produce a simpler, easily performed test would be desirable for future investigation.

### 6.3. Suggested Molecular Mechanisms

The molecular mechanisms by which a chronic asymptomatic *C. pneumoniae *infection might contribute to atherogenesis and lesional complications remain obscure. Since chlamydial organisms can infect and proliferate in cardiac tissues, a logical extension would be that Chlamydia could also infect the tissues of the coronary arteries, such as the endothelial and smooth muscle cells of these vessels.

Systemic dissemination of *C. pneumoniae *from the respiratory tract to the cells of the vascular wall requires a cellular transport system because Chlamydia replicate exclusively within their host cells. The elementary body, the metabolically inactive extracellular life stage of Chlamydia, has never been found circulating free within the bloodstream. Circulating monocytes, which are pivotal to the development of atherosclerosis, are known to migrate into the vascular wall. Recent studies have described the *in vitro* infection of monocyte-derived macrophages with *C. pneumoniae *and the presence of chlamydial DNA within peripheral blood mononuclear cells from patients with CAD [47, 48].

Atherosclerosis and chlamydial diseases share some interesting similarities, including the fact that they are chronic, subclinical, and inflammatory in nature [49, 50], with macrophage activation playing a key role in the inflammatory process [51]. Formation of macrophage foam cells in the arterial intima is the hallmark of early lesions in atherosclerosis [49], and the pivotal step in foam cell formation is the uptake of excess cholesterol from LDL [52]. *C. pneumoniae *was shown to induce macrophage foam cell formation in the presence of exogenous LDL [53], suggesting a causal role for the organism in atherogenesis. Kalayoglu and Byrne used the well-characterized murine macrophage cell line RAW-264.7 to examine the role of *C. pneumoniae *in foam cell formation by macrophages and showed that LPS is a *C. pneumoniae *component that induces macrophage foam cell formation in the presence of exogenous LDL and suggest that infected macrophages chronically exposed to *C. pneumoniae *lipopolysacharride (cLPS) may accumulate excess cholesterol to contribute to atheroma development [54].

Although endotoxin-mediated foam cell formation is not restricted to chlamydial LPS, it is improbable that other gram-negative organisms can invade and survive within the arterial intima to provide a continual source of antigen necessary to induce chronic inflammation and foam cell formation; unlike other gram-negative bacteria, *C. pneumoniae *has been detected within and isolated from atheromas [55–57], and it can survive and multiply within all cell types found in the atheroma.

It should be noted that acute microbial infections are known to affect the lipid metabolism in both experimental animals and humans. Profound changes in serum lipids are seen in acute pneumonia caused by *C. pneumoniae*; triglyceride concentrations are clearly elevated and HDL cholesterol levels decreased compared with the values in patients with pneumonia caused by other bacteria or viruses [58]. In a cross-sectional study, a significant association showed between the presence of *C. pneumonia* specific IgG antibodies, elevated serum triglyceride, and lowered HDL cholesterol concentrations in a male population in Northern Finland [59].

In another study, the serum triglyceride and total cholesterol concentrations were higher in the subjects with a chronic *C. pneumoniae *infection than in the subjects with no antibodies [36]. The HDL cholesterol concentrations and the ratios of HDL cholesterol to total cholesterol were significantly decreased in the subjects with chronic infection [36]. As it is noted before, chronic *C. pneumoniae *infection seems to be associated with a serum lipid profile considered to increase the risk of atherosclerosis.

Infections caused by other gram-negative bacteria have also been shown especially to affect triglyceride and HDL cholesterol levels. The phenomenon has been attributed to the action of lipopolysaccharide (LPS), an endotoxin that is a typical constituent of a gram-negative cell wall. LPS is an efficient inducer of several cytokines, for example, tumor necrosis factor-*α* (TNF-*α*). Administration of LPS, which mimics infection or TNF-*α* in both primates and Syrian hamsters, causes a decline of HDL cholesterol levels [60, 61], which could be attributed to a decrease in plasma lecithin: cholesterol acyltransferase activity [62, 63]. Furthermore, administration of TNF-*α* or interleukin-1 (IL-1) results in a rapid elevation of serum triglyceride levels followed by a later rise in cholesterol levels [64, 65]. The cytokine- and LPS-induced alterations in lipid metabolisms can be considered to be part of the acute-phase response and thus also beneficial to the host.

In addition, chlamydial LPS is the possible candidates responsible for the induction of inducible factors in human umbilical vein endothelial cells (HUVEC) including, components of the bacterial outer membrane complex, and heat shock protein 60 (HSP-60), which are known to be highly immunogenic. HSP-60 has recently been shown to induce cytokine and adhesion molecule expression in HUVEC [66] and has been found to colocalize with human HSP-60 in lesions of atherosclerosis [67]. The induction of smooth muscle cell (SMC) growth factor(s) by *C. pneumoniae*-infected endothelial cells represents a novel mechanism by which this bacterium may contribute to the immunopathogenesis of atherosclerosis.

So, the ability of *C. pneumoniae *to elicit an endothelial cell-derived soluble factor(s) that stimulates smooth muscle cells proliferation may be important in the pathogenesis of atherosclerosis. Several groups have demonstrated the ability of *C. pneumonia *to infect and replicate in cell types found within the atherosclerotic lesion, including endothelial cells, SMC, and macrophages [68, 69]. Infection of these cell types has been shown to result in the production of proinflammatory cytokines which may be involved in atherogenesis. The ability of Chlamydia to persist within host cells and produce antigens in the absence of replication may provide sustained immunogenic stimulation necessary for the development of chronic inflammatory diseases such as atherosclerosis. *C. pneumoniae *infection of endothelial cells has been shown to upregulate the expression of endothelial adhesion molecules [70] and several inflammatory mediators, including monocyte chemoattractant protein 1 (MCP-1), interleukin 8 (IL-8) [71], and IL-1b [72]. During atherosclerosis, these cytokines are also upregulated [49] and may potentiate the development of atheromatous lesions.

During the usual infective cycle generating new infectious progeny, Chlamydiae express basal levels of two major antigens: the major outer membrane protein (MOMP) and the heat shock protein 60 (HSP 60) [67]. Heat shock proteins (Hsps) belong to a family of approximately two dozen proteins whose amino acid sequences are highly homologous between widely divergent species, from bacteria to humans, and function to protect other proteins from denaturation. Because of this high degree of homology, there is a risk of immunological cross-reactions between microorganisms and vascular autoantigens. Hsps are subdivided into multimember families on the basis of the molecular weights of the proteins encoded (e.g., Hsp10, Hsp60, and Hsp70). HSPs (or chaperonins) are generally considered to act intracellularly to preserve cellular protein stability in response to conditions such as heat shock, nutrient deprivation, infections, and inflammatory reactions.

During chlamydial chronic, persistent infections, HSP 60 production increases substantially, whereas MOMP becomes almost undetectable [73]. Microbial HSP 60, abundantly produced during a chronic chlamydial infection of the vessel wall, might augment atherosclerosis and/or stimulate humoral and cellular immunity in atheroma. HSPs have been implicated as antigens stimulating autoimmunity in atherogenesis [74, 75]. In addition, bacterial HSP 60, like another microbial product such as LPS, may activate the innate immune system. Because human atheroma contains both human and chlamydial HSP 60s [13] and HSP 60s from different species share a substantial sequence similarity (the homologic results between the amino acid sequence of the chlamydial HSP-60 fragment and the corresponding human HSP-60 fragment is about 50%.), chlamydial and human HSP 60s might have similar functions on human vascular cells.

The homology between human and chlamydial HSP 60 suggests the possibility of antigenic mimicry. Chronic infection with *C. pneumoniae*, through the expression of HSP 60, might provoke an autoimmune reaction against human HSP 60. Indeed, patients with carotid atherosclerosis or coronary artery disease have high titers of antibodies against human HSP 60 [76, 77]. Human HSP 60 shares with the chlamydial protein have the ability to stimulate TNF-*α* and MMP-9 production by macrophages. When endothelial cells or macrophages express HSP 60 on their surface and are exposed to antibodies against HSP 60, they are susceptible to complement-mediated or antibodydependent cellular cytotoxicity [78–80]. If this were the case *in vivo*, this mechanism of cell injury might contribute to the pathobiology of atherogenesis.

A recent autopsy study showed greater frequency of chlamydial antigens in the cardiovascular tissue of patients who died of ischemic heart disease than in patients who died of noncardiac causes (64% versus 38%) [81].

Kol et al. demonstrated that chlamydial HSP 60 frequently produces in large amounts during chronic chlamydial infections and colocalizes with human HSP 60 in plaque macrophages in human atherosclerotic lesions. Chlamydial and human HSP 60 induce TNF-*α* and MMP production by macrophages. Chlamydial HSP 60 might mediate the induction of these effects by *C. pneumoniae*. Induction of such macrophage functions provides potential mechanisms by which chlamydial infections may promote atherogenesis and precipitate acute ischemic events [67].

 Now, it is known that *C. pneumoniae *can infect human endothelial cells (ECs) [82], where it induces the expression of adhesion molecules like endothelial-leukocyte adhesion molecule-1 (E selectin), intercellular adhesion molecule-1 (ICAM-1), and vascular cell adhesion molecule-1 (VCAM-1) [70]. *C. pneumoniae *also infects human monocyte—derived macrophages, stimulating the production of proinflammatory cytokines such as TNF-*α* and interleukin-6 (IL-6) [83]. The induction of adhesion molecules on infected endothelium might aid trafficking of monocytes and lymphocytes in atheroma [84, 85]. The induction of proinflammatory cytokines such as TNF-*α* or IL-6 might promote plaque instability and thrombosis [86, 87].

Because HSPs usually localize within cells, they require release into the extracellular space to activate vascular cells. In this regard, it is well known that Chlamydia, during their life cycle, undergo both phases of chronic, persistent, nonlytic infection, in which they remain viable but do not replicate, and phases of lytic infection [88]. During these lytic phases, the host cells release both their own HSP 60, produced during the previous chronic phase of infection, and also the human HSP 60, which has been produced in the host cell in response to the infection and to previous noninfectious stimuli [89], In addition, several observations support the occurrence of cell death within atheroma [90, 91], providing another pathway for release of HSPs from cells.

Kol et al. demonstrated that either chlamydial or human HSP 60 activates human vascular cell functions relevant to atherogenesis and lesional complications [90]. These findings contribute to the understanding of the molecular mechanisms by which a chronic asymptomatic chlamydial infection might influence atherogenesis and trigger acute events.

On the other hand, atheroma is an inflammatory site where a variety of cells, cell products, and lipoproteins interact to promote injury and disease [49]. An important consequence of these interactions is the cellular oxidation of low-density lipoprotein (LDL), which alters the lipoprotein to a highly atherogenic form [91, 92]. A variety of cell types present in atherosclerotic lesions, including monocytes/macrophages, smooth muscle cells, and endothelial cells, can oxidize LDL [92]. In turn, oxidized LDL promotes cell injury, smooth muscle cell proliferation, foam cell formation, chemotaxis of leukocytes, cellular secretion of inflammatory mediators, and other events that modulate atheroma biology [93–96]. Oxidized LDL has been detected in atheromas of rabbits and humans, and antioxidant therapy may decrease cardiovascular events and mortality [97, 98], perhaps by inhibiting the oxidation of lipoproteins. Therefore, oxidation of LDL, a biologically plausible mechanism of LDL modification, may explain why high plasma levels of native LDL are a major risk factor for coronary artery disease.

Infectious agents serve as a link between high serum LDL levels and an event critical to the development of the atheroma, such as the cellular oxidation of LDL. Oxidized LDL displays multiple atherogenic properties, including dysregulation of vascular tone, injury to the endothelium, promotion of leukocyte entry into the vessel wall, smooth muscle cell migration and proliferation, and foam cell formation [95–97, 99, 100]. Although serum antioxidants protect native LDL from oxidation in the macrovasculature, the atherosclerotic lesion contains oxidized LDL and may permit oxidation by acting as a sequestered microenvironment [92].

Although the mechanism by which *Chlamydiae *inhibit the respiratory burst remains unclear, such inhibition clearly favors survival for an obligate intracellular pathogen. Of interest, Chlamydiae can induce a selective release of myeloperoxidase without promoting superoxide production [101]. This observation may be relevant to *C. pneumonia*-induced monocyte activation, as myeloperoxidase has been proposed to play an important role in the oxidation of LDL *in vivo* [102]. Other potential mechanisms for *C. pneumoniae*-mediated macrophage LDL oxidation include induction of lipoxygenase [103], NADPH oxidase [104], thiol recycling [105], and transitional metal ions [106]. Monocyte-mediated LDL oxidation also may involve an increase in intracellular calcium and the activation of protein kinase C [107].

*Chlamydiophila pneumoniae *and macrophages are present in inflammatory tissue sites such as atherosclerotic lesions, where abnormal degradation of the extracellular matrix takes place. Vehmaan-Kreula et al. reported that production of 92 kDa gelatinase by human macrophages is selectively upregulated by *C. pneumonia* [14], which suggests that these bacteria, when present in a macrophage-containing inflammatory environment, actively participate in the destruction of the extracellular matrix. Matrix metalloproteinases (MMPs) are structurally related and participate in the degradation of extracellular matrix components. Besides participating in normal homeodynamics and developmental remodeling of connective tissues, the MMPs appear to contribute significantly, by their proteolytic activity, to the tissue damage seen in chronic inflammatory diseases such as rheumatoid arthritis, osteoarthritis, and atherosclerosis.

 Actually plaque rupture and increased thrombogenicity are prime mechanisms of acute coronary disease (ACD) and chronic *C. pneumonia *infection is linked to the precipitation of ACD. Dechend et al. examined whether infections with a *C. pneumoniae *strain isolated from such a coronary plaque would increase the expression of prothrombotic proteins in vascular cells. They found induced and sustained expression of functionally active TF and PAI-1 [16]. In addition to an increased procoagulation protein expression, functional cytokines are expressed in human arteriosclerotic lesions. IL-6 is an important mediator of inflammation in cardiovascular tissue [108]. IL-6 is highly expressed in arteriosclerotic lesions [109], implicated in plaque instability [110], and in the pathogenesis of acute myocardial infarction [108]. *C. pneumonia *infection induced sustained cellular overexpression and secretion of IL-6.

Recruitment of mesenchymal and immunocompetent cells, proliferation, and migration of VSMCs are the consequences of cytokine overexpression in the arteriosclerotic plaque [111]. This state of affairs further perturbs the anticoagulant activities. The functional cooperation between products of the coagulation cascade and cytokine-mediated inflammatory response has been shown to transform a stable plaque into an unstable plaque [111].

## 7. Conclusion

Atherosclerosis develops as a response of the vessel wall to injury. Careful review of epidemiological studies indicates that the classic risk factors, for example, hypercholesterolemia, cigarette smoking, and hypertension account for the majority but not the entirety of the etiology and pathogenesis of the clinical complications of atherosclerosis, including ischemic heart disease and acute myocardial infarction. Many underlying mechanisms that infectious diseases cause atherosclerosis have been proposed; most of them suggested that inflammatory process provoked by infectious diseases led to atherosclerosis. The macrophage is a critical component in the pathway to atherosclerotic inflammation. Several clinical studies were also performed to examine whether treatment for infectious diseases could protect against atherosclerosis. Given the burden that coronary artery disease imparts on the healthcare system and on society in general, efforts to both understand the role of infection in atherogenesis and to develop targeted intervention strategies should continue apace. However, the relationship between infectious diseases and atherosclerotic diseases and the effects of treatment for infectious diseases on atherosclerosis are still controversial; there are both positive and negative reports. Thus, further studies are needed to clarify the correlation.
